# Past President’s address

**DOI:** 10.4103/0019-5049.76560

**Published:** 2011

**Authors:** J Ranganathan

**Affiliations:** President, ISA National 2010 E-mail: drjrsalem@gmail.com

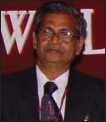



Respected Dr. A. P. Singhal, Past President of ISA, the Chief Guest of today’s function,

Dr. R. K. Sharma, Director, SGPGIMS, the Guest of Honour, Dr. (Brigadier) T. Prabakar, State President, UP & Uttarakhand, Dr. Virendrasingh, Secretary, and Dr. Sunil Saxena, President, Lucknow City Branch, Patron Dr. P. K. Singh, Dr. Surender Singh, Chairman, Organising Committee, Dr. Anil Agarwal, Organising Secretary, Dr. L. D. Mishra, Vice-President, ISA, Dr. S. S. Harsoor, Editor, IJA, Dr. Chakra Rao, Honorary Secretary, ISA, Dr. B. B. Mishra, Treasurer, ISA, and respectful past presidents, past secretaries, past treasurers, other office bearers, fellow members, invitees, press persons, special invitees Dr. Miller, Prof. R. K. Mirakhur, Dr. Manivannan and Dr. Makesh Vakamudi, Pharmaceutical and Equipment Companies, Ladies and Gentlemen,

With folded hands, I am welcoming all of you for the inaugural function.

I am greatly honoured to hold the highest post in our society. It gives me a proud feeling to be the leader of 16,000 anaesthesiologists. In the last 60 years, our association has grown up to phenomenal heights and now standing on her feet with the largest membership of anaesthesiologists. I am also glad to announce that our society is the second largest society in the world. Even though we are having a three-tier system of National, State and Local Branch, we are not able to represent equally. So, this year, we are introducing regional system in our governing body, a long pending demand of our members. For the regional governing council member, nominations will be received in the month of July by the National Head Quarters and all the elections will be held later on, so that they can take over the office on 29^th^ December every year, along with the newly elected national office bearers.

## MEMBERSHIP FEE

New members have to pay the membership fee directly to the national body and their share will be distributed to the state and city branches from the national body periodically. All the branches should submit their bank account number with IFSC code for easy transfer of money.

## FAMILY BENEVOLANT FUND

For the benefit of anaesthesiologists’ families, we introduced the family benevolent fund. But unfortunately, at branch level, this is not promoted well. Now, we want to give the deceased anaesthetist’s family, a minimum of ` 15 lakhs. Hence, we want to introduce this to the new members for a period of 20 years. They have to pay the premium for a period of 20 years only. After the completion of 20 years, money will be given to the deceased anaesthesiologist’s family from the corpus fund. For the continuity of the service of the governing council, I opine that every year we have to elect the President Elect, who will be in the council. Next year, he will take over the charge as President, to maintain a better continuity of service to the society.

## MEDICAL EDUCATION

The uniformity of the standard of practice is not maintained in the country. Hence, ISA started Indian College of Anaesthesiologists 10 years back. I feel it is the right time to give adequate training and we can have uniform examination systems. In future, we are going to award ISA recognised fellowship certificates to them. This year, in November 2010, ICA conference was held at Rothak in Haryana. There, we requested the Chief Minister of Haryana to allot the land for Indian Society of Anaesthesiologists at Gurgaon, next to Delhi, which we could use for our college and also for our National Head Quarters’ office.

In the recent MCI declaration form for the medical teacher, diploma holder of anaesthesia is not considered in any of the faculty positions, even though the diploma qualification is awarded by MCI. With the qualification from MCI, they are not recognised as teachers. As such, 3 years teaching experience is required to become a teacher. Diploma courses are only for 2 years. Already ISA has represented to MCI and the Government of India, to conduct a short-term course for diploma holders in anaesthesia and award MD degree, so that their qualification isvalidated.

To meet the challenge of the shortage of anaesthesiologists in the country, we are proposing to convert all diploma seats to MD seats. By doing this, the shortage of teaching faculty can be rectified in due course and also rural people will get much better medical facility.

During the teaching course of MD, in the final year, at least 3–6 months posting can be given in the rural hospitals, along with one qualified anaesthetist. This would benefit the rural population also as they would get the services of qualified anaesthetist for the procedures like caesarean section and in trauma care – during the golden hour treatment.

Several of our city branches are conducting regular monthly meetings, continuing medical education programmes periodically and hands on workshops to update the knowledge of the anaesthesiologists.

ISA branches are conducting social awareness programme during World Anaesthesia Day on 16 October. These programmes generate public awareness through media about anaesthesia. Now, our branches with the help of National Head Quarters are actively conducting Disaster Management Programme and Highway Rescue Project on National Highways for the trauma victims. Throughout the year, they are conducting basic life support training for different levels of population like school children, factory workers, transport drivers and police and fire brigade people, etc.

We want to update the election process through electronic voting system. For that, on World Anaesthesia Day (16 October), we tried the mock election. Unfortunately, out of 15,000 members, only a few votes were polled. Hence, electronic voting system appears to be a premature one. But we can use electronic media for opinion poll probably for this year. We can try it in the national conference and also in the regional conferences. If it is successful, we can try electronic voting system after 2 or 3 years.

We are running our journal *IJA* successfully without any delay for the past 5 years. But the only problem is that whenever there is a change of editor, the system collapses for 6 months. At least for two to three issues, we are paying the full postages, which come to around ` 25–30 per copy. This collapse happens only due to inadequate exchange of information between the new editor and past editor. Also, the interpretation of postage, which varies from place to place, is another cause. Hence, we are losing huge amount of money towards postage expenditure. So, we are planning an alternative system to improve the present setup.

The ISA is a founder member of World Federation of Anaesthesia. Now, there is a problem that for annual subscription, an amount of ` 17–20 lakhs is required every year towards the payment for 16,000 members, whereas presently we are only paying for 1101 members at $2.5 for each member annually. Though we are collecting one time Lifetime subscription payment for our memberships, the WFSA membership needs to be paid on an annual basis. I am hopeful that in our General Body, we will sort out the issue amicably.

We conducted eight ISA sponsored CME programmes this year from Assam to Gujarat, and from Kanyakumari to Saranath, and one more will be conducted in the month of January.

This year, all the national office bearers attended many of the state and zonal conferences. During this, we are able to understand that most of the branches are conducting meetings, but they are not reporting to state or national body. This sort of practice should be avoided. Because an individual is not superior and always society is superior, this will help you in distress. Each one, Catch one!

Finally, I request the medical persons present here to enlighten the public about anaesthesia, our life-saving ability, critical care and also pain management. Even though we are doing great amount of service within four walls of operation theatre, it is never highlighted. We are the pain relievers for the patient as well as saviors to the society.

Wish you happy and prosperous and peaceful 2011.

Long Live ISA!
Jai Hind!

